# Secondhand smoke exposure, diabetes, and high BMI are risk factors for uterine cervical cancer: a cross-sectional study from the Korea national health and nutrition examination survey (2010–2018)

**DOI:** 10.1186/s12885-021-08580-3

**Published:** 2021-07-31

**Authors:** Ji Young Kim, Dae Woo Lee, Min Jeong Kim, Jae Eun Shin, Yeun Joo Shin, Hae Nam Lee

**Affiliations:** Department of Obstetrics and Gynecology, Bucheon St, Mary’s Hospital, College of Medicine, The Catholic University of Korea, 327, Sosa-ro, Bucheon-si, Gyeonggi-do, 14647 Seoul, Republic of Korea

**Keywords:** KNHANES (Korea National Health and nutrition examination survey), Cervical cancer, Diabetes, BMI, Secondhand cigarette smoke

## Abstract

**Background:**

Human papilloma virus infection and tobacco smoking are the major risk factors for cervical cancer. There are limited studies searching other risk factors for cervical cancer and the results are not consistent. This study investigated the relations between cervical cancer and possible risk factors, including secondhand cigarette smoke exposure, diabetes, body mass index (BMI), and work schedule.

**Methods:**

In this cross-sectional study, 29,557 women completed a cervical cancer questionnaire and were selected using 2010–2018 data from the Korea National Health and Nutrition Examination Survey. Details in secondhand smoke exposure, diabetes, BMI, and work schedule were assessed with participants’ health interviews and health-related surveys.

**Results:**

Two hundred sixty-two women (0.89%) in the sample were diagnosed with cervical cancer. Domestic secondhand smoke exposure, diabetes, and high BMI significantly increased cervical cancer risk. Respective odds ratios and 95% confidence intervals were: 1.547 (1.042–2.297), 2.156 (1.535–3.027), and 1.036 (1.006–1.067). Weekly work hours, and work schedule were not significantly related to cervical cancer incidence.

**Conclusion:**

Among Korean women, passive exposure to cigarette smoke at home, diabetes, and high BMI increase risk for cervical cancer.

## Background

Cervical cancer is the fourth most common and the third most lethal female malignancy worldwide [1]. Cervical cancer is caused by human papilloma virus (HPV) infection, which is one of the most powerful human carcinogens and has been implicated in several cancers, including uterine cervix, anorectum and oropharynx [2]. In addition to HPV infection, young age at first intercourse, multiple sexual partners, cigarette smoking, race, high parity, low socioeconomic status, and chronic immune suppression are also risk factors for cervical cancer [3].

Tobacco smoking is a strong risk factor for cervical neoplasia. Smoking status, duration, and intensity are associated with twofold increase in risk for high-grade cervical dysplasia and invasive carcinoma, independent of HPV infection [4]. Passive cigarette smoking, or secondhand smoke exposure is also considered a risk factor for cervical carcinogenesis, although related study results have been inconsistent. Su et al. reported a 1.7-fold increase in cervical cancer risk among those exposed to secondhand smoke, whereas Louie et al. concluded that passive smoking is not an independent risk factor for invasive cervical cancer in the absence of active smoking [5, 6].

Diabetes mellitus (DM), especially type 2 diabetes, is a major risk factor for many cancers. The association between endometrial cancer and diabetes is well-established, and some have shown weak correlations between ovarian cancer and diabetes [7, 8]. However, the relationship between cervical cancer incidence and type 2 diabetes remains unclear and relevant studies have been limited.

Increased body mass index (BMI) has been considered to increase the risk of many cancers. One meta-analysis reported that each 5 kg increase in adult weight gain raises the risk of post-menopausal breast (11%), endometrial (39%) and ovarian cancer (13%) [9]. A longitudinal study from the United States suggested that a longer duration of overweight and obesity is associated with an increased risk of developing several forms of cancer, such as breast, colon, endometrial, and kidney cancer [10]. Not many studies are there for the association between obesity and cervical cancer.

Socioeconomic disparities among women also affect cervical cancer occurrence. Cervical cancer incidence and mortality are much higher in low- and middle-income countries compared with high-income countries [11]. In Korea, women with lower education levels and lower household income have significantly lower cervical cancer screening rates than do highly educated and high-earning females [12, 13]. Regarding occupation, service and sales workers appear to have a higher risk of cervical cancer compared with those in other fields [14].. To our knowledge, the relationship between work schedule and cervical neoplasm has not been addressed.

To better prevent cervical cancer, and raise public health awareness, risk factors other than HPV infection and smoking must be identified. Thus, the study purpose was to determine the relationships between cervical cancer and potential risk factors, including passive cigarette smoking, diabetes-related factors and work schedule, using data from the Korea National Health and Nutrition Examination Survey (KNHANES).

## Methods

The analyses presented herein are based on data collected during the 2010–2018 KNHANES, which began in 1998 and is administered by the Division of Health and Nutrition Survey under the Korea Disease Control and Prevention Agency. KNHANES is an ongoing population-based cross-sectional survey designed to assess the health and nutritional status of Koreans. The study monitors trends in health risk factors, assesses the prevalence of major chronic diseases, and provides data for the development and evaluation of health policies and programs in Korea [15].

The health interviews and examinations were conducted in mobile examination centers, while the nutritional surveys were performed by trained physicians or nurses who visited each household. Participants were asked to complete the cervical cancer, diabetes, smoking, and working conditions questionnaire during their health interviews. Written informed consent was acquired from all participants before the survey was administered. Institutional Review Board of the Catholic University of Korea, Bucheon, Republic of Korea approved this study (HC20ZASI0107).

During the 2010–2018 KNHANES, 32,485 women aged 18 years or older participated. The annual participant distribution is shown in Table 1. Among the 29,557 women who completed the cervical cancer questionnaire, 262 (0.89%) were diagnosed with cervical cancer. Among the sample with cervical cancer, there were 45 women with diabetes, who were classified into three groups according to treatment method: insulin injection, medication, or nonpharmacological treatment. Participants who reported secondhand smoke exposure were asked to identify whether this occurred in their workplace, home, or public places. Work schedule was categorized as: day shift, evening shift, night shift, regular 12-h shift, 24-h shift, split shift, irregular shift, or other. Having cervical cancer or diabetes at the health interview was defined based on a diagnosis by a qualified physician.

**Table 1:** Sample distribution by study year

Year	Without cervical cancer (n)	Cervical cancer (n)	Cervical cancer (%)
2010	3555	30	0.84
2011	3478	23	0.66
2012	3313	16	0.48
2013	3103	33	1.06
2014	2942	31	1.05
2015	2921	27	0.92
2016	3337	38	1.14
2017	3252	36	1.11
2018	3394	28	0.82
total	29,295	262	0.89

Statistical analyses conducted using Stata (v. 16.1; StataCorp LLC, College Station, TX, USA), reflect the complex sampling design and sampling weights of the KNHANES, and were chosen to provide nationally representative prevalence estimates. Fisher’s exact probability tests were performed to identify distribution patterns by cervical cancer occurrence and odds ratios (OR), along with 95% confidence intervals (CI), were calculated using logistic regression models with respective independent variables. Adjusted ORs with 95% CIs were calculated using multiple logistic regression analysis, including variables that were statistically significant on univariate analysis. To compare mean BMI and weekly work hours, independent samples t-tests were applied.

## Results

Distribution patterns by categories are shown in Table 2. To investigate the relations between passive smoke exposure and cervical cancer occurrence, secondhand smoke in the workplace, home, and public places were analyzed separately. Passive smoke exposure at work was negatively related to cervical cancer risk. Incidence of cervical cancer among women exposed to secondhand smoking at work was 0.57%, whereas 0.95% of unexposed women had cervical cancer (*χ*2 = 6.835, *p* = 0.009). Domestic passive smoke exposure was related with increased cervical cancer incidence. Cervical cancer was present among 1.26% of women exposed domestic secondhand smoke, but only 0.85% of those living in a smoke-free home (*χ*2 = 4.017, *p* = 0.045). Among women exposed to secondhand smoke in public places, 0.84% had cervical cancer, which did not differ significantly from the rate of 1.01% among those who were unexposed (*χ*2 = 0.569, *p* > 0.05).

**Table 2:** Sample distribution by passive smoking, diabetes, DM treatments, and work schedule

Variables			n	Cervical cancer (%)	p^*^
Passive smoking	Workplace	Unexposed	24,044	228 (0.95)	0.009
		Exposed	5081	29 (0.57)	
	Domestic	Unexposed	27,142	230 (0.85)	0.045
		Exposed	2222	28 (1.26)	
	Public place	Unexposed	10,680	108 (1.01)	0.451
		Exposed	2264	19 (0.84)	
Diabetes		Nondiabetic	27,175	217 (0.80)	0.000
		Diabetic	2380	45 (1.89)	
DM treatment	Insulin injection.	No	29,340	258 (0.88)	0.124
		Yes	214	4 (1.87)	
	Medication	No	27,427	219 (0.80)	0.000
		Yes	2127	43 (2.02)	
	Nonpharmacologic	No	29,247	256 (0.88)	0.045
		Yes	307	6 (1.95)	
Work schedule		Day shift	13,973	100 (0.72)	0.354
		Evening shift	2102	16 (0.76)	
		Night shift	278	0 (0.00)	
		12-h regular shift	297	0 (0.00)	0.438
		24-h regular shift	43	0 (0.00)	
		Split shift	133	1 (0.75)	
		Irregular shift	93	1 (1.08)	
		Other	64	0 (0.00)	

^*^p value calculated by Fisher’s exact test

*DM* diabetes mellitus

Using pooled data, cervical cancer prevalence among women with diabetes was 1.89%, significantly higher than 0.80% among women without diabetes (*χ*2 = 29.712, *p* < 0.001). However, insulin usage for diabetes treatment was not significantly related to cervical cancer occurrence (*χ*2 = 2.369, *p* > 0.05). Nonpharmacologic DM treatment (*χ*2 = 4.026, *p* = 0.045) and medication prescribed for diabetes (*χ*2 = 33.610, *p* < 0.001) were significantly related to cervical cancer. Mean BMI among those with cervical cancer (24.22 ± 3.53) was substantially higher than among those without cervical cancer (23.47 ± 3.58; *t* = − 3.403, *p* = 0.001).

Mean weekly work hours among women with cervical cancer (34.74 ± 16.69) did not differ significantly from women without cervical cancer (37.21 ± 18.35; t = 1.460, *p* > 0.05). Nor were there difference between those working the day, evening, or night shifts (χ2 = 2.074, *p* > 0.05) or between those working regular 12-h, 24-h, split, irregular, or another shift type (χ2 = 3.767, *p* > 0.05).

The relationships between cervical cancer and passive smoking, diabetes, BMI, weekly work hours, and work schedule are shown in Table 3. The OR for cervical cancer based on exposure to secondhand smoke at home was 1.488 (95% CI: 1.002–2.207, *p* = 0.049). For exposure to secondhand smoke in the workplace, the OR for cervical cancer was 0.595 (95% CI: 0.404–0.876, *p* = 0.009). In multiple logistic regression analysis after adjusting for other parameters which were statistically significant in the univariate analysis, the OR for cervical cancer with domestic secondhand smoke exposure was 1.547 (95% CI: 1.042–2.297, *p* = 0.03). For exposure to workplace secondhand smoke, the adjusted OR was 0.603 (95% CI: 0.408–0.891, *p* = 0.011).

**Table 3:** Relations between risk factors and cervical cancer

		Univariate		Multivariate	
		OR^a^ (95% CI)	*p*	Adjusted OR^b^ (95% CI)	*p*
Passive smoking	Work place	0.595 (0.404–0.876)	0.009	0.603 (0.408–0.891)	0.011
	Domestic	1.488 (1.002–2.207)	0.049	1.547 (1.042–2.297)	0.030
	Public place	0.836 (0.513–1.365)	0.475		
Diabetes		2.369 (1.713–3.274)	0.000	2.156 (1.535–3.027)	0.000
BMI		1.056 (1.026–1.086)	0.000	1.036 (1.006–1.067)	0.020
Weekly work hours		0.992 (0.983–1.002)	0.100		
Working pattern	Night shift	1.093 (0.644–1.857)	0.741		
	Split shift	0.689 (0.042–11.234)	0.794		

^a^OR calculated using logistic regression test, ^b^Adjusted OR calculated using multiple logistic regression test

*CI* confidence interval; *DM* diabetes mellitus; *BMI* body mass index; *OR* odd ratio

The relation between diabetes and cervical cancer was significant (OR: 2.369, 95% CI: 1.713–3.274, *p* < 0.001). After adjusting for other variables, diabetes still showed a statistically significant association with cervical cancer (adjusted OR: 2.156, 95% CI: 1.535–3.027, *p* < 0.001).

BMI level (OR: 1.056, 95% CI: 1.026–1.086, *p* < 0.001) was positively related to cervical cancer incidence. In multivariate analysis BMI still showed significant association with cervical cancer (Adjusted OR: 1.036, 95% CI: 1.006–1.067, *p* = 0.020).

Regarding weekly work hours and work schedule, insulin usage and non-pharmacological method for DM treatment, there were fewer than expected participants in many cells, which thus failed to meet the analysis criteria. Consequently, the omnibus analyses for these factors were not statistically significant.

## Discussion

This study investigated whether secondhand smoke exposure, diabetes, BMI, or work schedule are risk factors for cervical carcinogenesis.

The role of passive cigarette smoking in cervical neoplasia is controversial. Herein, secondhand smoke exposure at home was significantly related to cervical cancer. This finding is consistent with most studies, which have shown that secondhand smoke exposure is positively correlated with cervical cancer [5, 16–18]. Some studies have also reported passive smoking as an independent risk factor for abnormal cervical cytology or cervical intraepithelial neoplasm (CIN) [19–21]. However, most reports did not adjust for covariates like dose of tobacco exposure, sexual behavior, socioeconomic conditions, and (especially important) HPV infection status. A few investigators have proposed that passive smoking is statistically unrelated to CIN or cervical cancer after accounting for HPV infection status [6, 22, 23]. Interestingly, we identified that workplace secondhand smoke exposure is associated with lower cervical cancer risk. Considering the KNHANES data characteristics, it is possible that women diagnosed with cervical cancer may seek out smoke-free work environment and move to such jobs.

There was a substantial association between diabetes and cervical cancer. Several studies have reported increased risk for and mortality from many cancer types among patients with diabetes, especially type 2 DM [7, 8, 24–26]. Some characteristics of diabetes may explain this carcinogenic tendency, including hyperinsulinemia, hyperglycemia, and chronic inflammatory status. These conditions all encourage cellular proliferative, angiogenetic, antiapoptotic, and metastatic activities [7, 27]. After adjusting for other variables, the association between diabetes and cervical cancer was still markedly increased and statistically significant. Regarding oral antidiabetic agent, researchers have suggested that metformin therapy lowers cancer risk, while sulfonylureas increase carcinogenesis risk [28–30]. Herein, use of antidiabetic drugs increased cervical cancer risk. Because these data did not include which diabetic drugs were used, it is difficult to either determine the effects of individual drugs or interpret these findings more generally. Moreover, because diabetic drugs are almost exclusively given to DM patients and those factors are highly correlated, the DM medication was excluded from the multiple logistic regression model.

BMI was positively associated with cervical cancer not only independently, but also after adjusting for other factors. Some researchers have also reported that obesity is weakly associated with an increased risk of cervical cancer [31]. One interesting study showed that obese women are at increased risk of having inappropriate cervical cancer screenings before cancer diagnosis, possibly due to a negative body image, bias on the part of health care providers, poor health behaviors, and comorbidities affecting regular care [32].

No notable results were found regarding work hours and schedule. Few studies have explored the potential associations between night shift work and cancer risk, reporting lack of evidence or few correlations [33, 34]. Since our data were cross-sectional, it is difficult to suggest causal relations. For example, women who were diagnosed with cervical cancer may have reduced their work hours or moved to a shift that carried fewer burdens. Further prospective studies are needed to assess a broad range of occupations and various socioeconomic characteristics.

A major strength of this study is that it was based on extensive nationwide survey data, including 29,557 women in Korea. Furthermore, relatively recent data (2010–2018) were used, reflecting current disease patterns and trends. There are also several limitations. The study was cross-sectional; thus, exposure and outcomes were assessed simultaneously and deductions about interrelated courses are difficult. Finally, most of these data were derived from self-report questionnaires based on participant recall.

## Conclusion

Several factors, including passive cigarette smoking at home, diabetes, and high BMI, are related to increased risk of cervical cancer among women in South Korea.

## Data Availability

The datasets analyzed during the current study are available in website of The Korea Disease Control and Prevention Agency; Korea National Health and Nutrition Examination Survey repository, [https://knhanes.cdc.go.kr/knhanes/sub03/sub03_02_05.do].

## Abbreviations

BMI: Body mass index
OR: Odds ratio
CI: Confidence intervals
HPV: Human papilloma virus
DM: Diabetes mellitus
KNHANES: Korea National Health and Nutrition Examination Survey
CIN: Cervical intraepithelial neoplasm
